# Acceptability of a long‐acting injectable HIV prevention product among US and African women: findings from a phase 2 clinical Trial (HPTN 076)

**DOI:** 10.1002/jia2.25408

**Published:** 2019-10-25

**Authors:** Elizabeth E Tolley, Sue Li, Sahar Z Zangeneh, Millicent Atujuna, Petina Musara, Jessica Justman, Subash Pathak, Linda‐Gail Bekker, Shobha Swaminathan, Jill Stanton, Jennifer Farrior, Nirupama Sista

**Affiliations:** ^1^ FHI 360 Durham NC USA; ^2^ Vaccine and Infectious Disease Division Fred Hutch Seattle WA USA; ^3^ Desmond Tutu HIV Centre University of Cape Town Cape Town South Africa; ^4^ University of Zimbabwe Harare Zimbabwe; ^5^ Columbia University New York NY USA; ^6^ Rutgers New Jersey Medical School Newark NJ USA

**Keywords:** HIV prevention, clinical trial, acceptability, PrEP, injectable, women

## Abstract

**Introduction:**

High HIV incidence and low adherence to daily oral PrEP among women underscore the need for more acceptable and easier to use HIV prevention products. Global demand for injectable contraception suggests that new, long‐acting, injectable formulations could meet this need. We examine acceptability of a long‐acting injectable PrEP among HIV‐uninfected women in Zimbabwe, South Africa and two United States phase 2 trial sites.

**Methods:**

Quantitative surveys were administered at the first, fourth and sixth injection visits. Focus group discussions (FGD) were conducted after the sixth injection visit. We compared the acceptability of injectable product attributes, prevention preferences and future interest in injectable PrEP by site and arm and ran longitudinal ordinal logistic regression models to identify determinants of future interest in injectable PrEP.

**Results:**

Between April 2015 and February 2017, the trial enrolled 136 (100 African, 36 US) women with a median age of 31 years. Most participants (>75%) rated injectable attributes as very acceptable. While few reported rash or other side effects, 56% to 67% reported injection pain, with nonsignificant differences over time and between arms. During FGDs, participants described initial fear of the injectable and variable experiences with pain. Most US and African participants preferred injectable PrEP to daily oral pills (56% to 96% vs. 4% to 25%). Future interest in using injectable PrEP was associated with acceptability of product attributes and was higher in African than US sites. In FGDs, participants described multiple reasons for trial participation, including a combination of monetary, health‐related and altruistic motivations. While associated with future interest in use in univariate models, neither altruistic nor personal motivations remained significant in the multivariate model.

**Conclusions:**

This study found that long‐acting injectable PrEP is acceptable among African and US women experiencing product use. Acceptability of product attributes better predicted future interest in injectable use than experience of pain. This is reassuring as a single‐dose regimen of a different product has advanced to phase 3 trials. Finally, the study suggests that future demand for an injectable PrEP by women may be greater in African than US settings, where the risk of HIV is highest.

## 1 Introduction

Globally, women account for almost half of the approximately 37 million people living with HIV/AIDS, ranging from 19% of new infections in the US to almost 60% in sub‐Saharan Africa (SSA) 1. While oral PrEP has demonstrated efficacy in women and is approved for use in the US and many countries in SSA, uptake remains low. Lack of awareness, low HIV risk perception and concerns about or challenges taking daily oral pills are key factors undermining use 2, 3, 4, 5, 6. The need for new HIV prevention methods that women find acceptable and easy to use continues to be pressing.

The pre‐exposure prophylactic (PrEP) use of daily oral antiretroviral drugs can dramatically reduce the risk of acquiring HIV. In a systematic review of 18 randomized controlled trials, open‐label extensions or demonstration studies, those achieving high levels of product adherence observed a 70% overall reduction in the risk of HIV infection 7. These studies targeted diverse populations, including HIV discordant couples 8, men‐who‐have‐sex‐with‐men (MSM) and transgender women 9, and heterosexual men and women 10; they also assessed different regimens and dosing strategies, including daily immediate or delayed dosing 11 or other intermittent dosing schemes 12. The systematic review suggests that the level of adherence achieved within the trial, rather than mode of transmission, sex of participant, PrEP regimens or dosing schemes was the primary moderator of PrEP effectiveness.

Although adherence is likely the key to achieving ARV‐based PrEP effectiveness, determinants of adherence have varied for different populations. Two large Phase 3 PrEP trials conducted with heterosexual African women failed to demonstrate effectiveness. In both trials, product adherence was low – especially in younger women, under the age of 25 13, 14, 15. While reporting moderate levels of effectiveness, two recent trials of PrEP vaginal rings noted similar challenges 16, 17. The wide variations in adherence achieved by women in different PrEP trials have been attributed to various factors including participants’ level of perceived HIV risk, the acceptability of product‐related attributes, women’s ability to disclose product use and/or trial participation to sexual partners or others, and their underlying motivations for trial participation 18, 19, 20.

Early findings from PrEP demonstration studies suggest that adherence to PrEP regimens may be easier when taking a product of known effectiveness 21, 22. Nevertheless, contraceptive research supports the notion that women’s preferences for method‐specific prevention strategies vary widely and adherence‐related challenges will differ by product 23. Continuing high HIV incidence among young women – especially in Sub‐Saharan Africa – and strong global demand for injectable contraception 24 suggest that newer, longer‐acting injectable (LAI) PrEP may help women meet this need for protection.

HPTN 076 was one of two Phase 2 trials testing the safety and tolerability of a LAI PrEP formulation. This randomized, controlled trial took place between April 2015 and February 2017 and compared the safety of TMC278 LA (rilpivirine) to inactive placebo administered every eight weeks over a forty‐week period for a total of six injections. The HPTN 076 trial provided an opportunity to examine and compare acceptability of a LAI PrEP product among HIV‐uninfected, low‐risk women, aged 18 to 45 years old, across four settings in Africa and the United States – Harare, Zimbabwe; Cape Town, South Africa; Newark, New Jersey and Bronx, New York.

In this study, we assessed acceptability within a multidimensional framework, determined not only by users’ perceptions of product attributes (e.g. location, size, number and frequency of injections), but also the perceived need for HIV prevention and product‐specific preferences outside of a trial context. We further theorized that women’s motivations for trial participation might lead them to join trials without a strong interest in using prevention in the future, and that product‐related preferences would be influenced by women’s familiarity with different product modalities.

## 2 Methods

Two types of data were collected to assess acceptability of TMC 278 LA. As part of their routine visits, all trial participants were administered quantitative surveys at enrolment, and after the first, fourth and sixth injection visits. In addition, a subset of participants from each site were invited to participate in one of three focus group discussions (FGDs).

### 2.1 Quantitative survey measures

Enrolment acceptability measures, collected at baseline only, examined women’s previous use of injections for contraception or other prevention/treatment purposes, HIV risk perception, past HIV prevention behaviours and initial preferences for future HIV prevention product use. We assessed women’s motivations for trial participation based on how important specific reasons were (1 = not at all important to 6 = very important) for joining the trial, including the “desire to help scientists find new HIV prevention methods” and “access to medical tests and procedures.” Similarly, acceptability of five product attributes and three physical experiences were measured on a scale of 1 = highly unacceptable to 6 = highly acceptable after the first, fourth and sixth injection. Product attributes included: receiving two injections at a visit; size/quantity of each injection; receiving injections every two months; injection site in the buttocks and degree of privacy. Physical experiences included: any pain at injection site; any rash/reaction at injection site; and any side effects experienced since last injection. If participants did not experience any pain, rash/reaction or side effects, their response was coded as 6 = highly acceptable.

We conducted separate principal component analyses (PCA) with varimax rotation on the set of seven items describing motivations for trial participation and the eight items describing acceptability of “injectable attributes” and “physical experiences.” The PCA of trial motivations showed two distinct PCs where the first PC comprised three items denoting “altruism” and the second PC comprised four items describing “personal and health benefits.” Similarly, two distinct PCs were found in the PCA of acceptability where the first PC comprised five product acceptability items denoted as “injectable attributes” and the second PC comprised three items denoted as “physical experiences.” The scores for each of the four PCs were calculated as weighted sums of measures of their components with weights $\left(W_{i}/\sum W\right)$, where w_i_s were the loadings from the principal component analysis. The ranges of scores were the same as the ranges of the measures of components 1, 2, 3, 4, 5, 6.

In addition to participants’ subjective attitudes towards physical experiences, injection site reactions (ISR) were recorded on an adverse event log by clinical staff at any clinic visit, when observed. We included a count variable for the total number of ISRs, regardless of grade, in our model. Finally, two outcome variables were considered for this analysis. The first assessed participants’ HIV prevention preferences; options included oral PrEP, vaginal rings or gel, and injectables or no preference. We also measured participants’ level of agreement to six statements to assess “future interest in injectable PrEP” (FIIP). HIV prevention preferences and FIIP were measured after the fourth and sixth injection visit. We used the most extreme, positive statement of the six, “You would definitely use the injection for some time” as our primary dependent variable.

### 2.2 Quantitative analyses

We compared baseline socio‐demographic and risk characteristics between US and Africa sites using Chi‐square or Fisher’s Exact tests for categorical variables and t‐tests for continuous variables. For prevention preferences, we compared the proportions of US versus Africa participants who preferred various prevention products within each visit (baseline and Week 28) and between two visits within US sites and Africa sites using Fisher’s Exact test.

We performed univariate and multivariate analyses using a longitudinal ordinal logistic regression model to identify determinants of FIIP. We considered the binary baseline covariates of “Ever injectable contraception,” “HIV risk perception” and two composite variables representing altruistic and personal motivations for trial participation. We also included numeric time‐varying covariates describing “Level of condom use last month” and summary scores describing acceptability of “injectable attributes” and “physical experiences.” Treatment arm and site (US vs. African) were also included in the model. The models were fit via generalized estimating equations (GEE), assuming an independence covariance structure 25, using Proc Genmod procedure in SAS. All analyses were implemented using SAS software (version 9.4).

### 2.3 Sensitivity analyses

Prior to final selection of our primary outcome variable, “Future interest in injectable PrEP” (FIIP), we fit a model with the positive statement, “You would definitely use the injection for some time” and then conducted a sensitivity analysis using the negative statement “You would not use the injection.” Because results from both regression models were qualitatively similar, we used the positive statement, measured at week 44. In addition, we examined the association between permanent product discontinuation (binary, time‐varying) and FIIP, using the same univariate modelling approach described above, to assess whether women who discontinued product use during the trial may have had lower acceptability than women who continued use.

### 2.4 Qualitative FGDs

Women still in follow‐up between weeks 44 and 76 were invited to participate in an FGD after moving off active product use. Three FGDs were conducted, one each in Cape Town, Harare and New York/New Jersey, with eight to 12 participants per FGD who were selected from a list of randomly sampled participant identification numbers provided to each site. The FGD explored the perceived need for an injectable HIV prevention method; trial and product‐related experiences, including the impact of partners, family members and others; and future interest in injectable PrEP. FGDs were conducted in the local language by interviewers trained in open‐ended interviewing techniques. The audio‐recordings were transcribed, translated into English and typed into word documents for analysis.

### 2.5 Qualitative analyses

We imported the FGD transcripts into NVivo version 10. Data were analysed by a team of two qualitative analysts who independently read the first transcript to identify emergent themes. They discussed and agreed on a simple codebook to identify thematic content related to HIV risk perception, motivations for and experiences with trial participation, attitudes towards injectable attributes, and perspectives on who might use an injectable and why. Subsequently the second analyst applied the codes, and both analysts developed thematic memos and data matrices 26 to identify and compare product and trial‐related attitudes and experiences across sites. The team reviewed and discussed these analytic products to ensure they had similar interpretations of the data. We incorporated the qualitative data to shed additional light on the survey findings.

### 2.6 Ethical statement

HPTN 076 was reviewed and approved by national and local ethics review committees in each site. All participants provided voluntary written informed consent to participate in the trial; FGD participants provided separate informed consent for this activity.

## 3 Results

The study enrolled 136 women (100 African, 36 US) with a median age of 31 years. Approximately half of participants were married, with the Zimbabwe site contributing most to this group. Two‐thirds of African participants and about half of US participants were unemployed. At baseline, more than half (57%) of participants in the African sites and 47% in the US sites reported ever using injectable contraception and just over half of ever users reported currently using injectable contraception (Table 1).

**Table 1:** Baseline socio‐demographic & risk characteristics

	Overall (n = 136)	US (n = 36)	Africa (n = 100)	*p*‐values
Arms assigned				
Placebo	33% (45/136)	33% (12/36)	33% (33/100)	n/a
Active	67% (91/136)	67% (24/36)	67% (67/100)	
Age				
Mean	31	33	31	0.20
Median (Q1, Q3)	31 (25, 38)	32 (28, 40)	31 (24, 37)	
Marital status				
Married/civil union	46% (63/136)	19% (7/36)	56% (56/100)	<0.001
Living with primary partner	5% (7/136)	19% (7/36)	0% (0/100)	
Primary partner, not living together	15% (21/136)	0% (0/36)	21% (21/100)	
Single/divorced/widowed	33% (45/136)	61% (22/36)	23% (23/100)	
Race				
Asian	1% (1/136)	3% (1/36)	0% (0/100)	<0.001
Black/African American	94% (128/136)	78% (28/36)	100% (100/100)	
Multiracial	1% (1/136)	3% (1/36)	0% (0/100)	
Other	2% (3/136)	8% (3/36)	0% (0/100)	
White	2% (3/136)	8% (3/36)	0% (0/100)	
Employment status				
Full‐time employment	17% (23/136)	33% (12/36)	11% (11/100)	0.009
Part‐time employment	23% (31/136)	19% (7/36)	24% (24/100)	
Not employed	60% (82/136)	47% (17/36)	65% (65/100)	
Personal benefits score				
Number of non‐missing values	136	36	100	0.001
Mean	4.8	4.2	5.0	
SD	1.2	1.2	1.2	
Min, Max	1.7, 6.0	2.0, 6.0	1.7, 6.0	
Altruism score				
Number of non‐missing values	135	36	99	<0.001
Mean	5.9	5.6	6.0	
SD	0.3	0.5	0.2	
Min, Max	4.1, 6.0	4.1, 6.0	4.6, 6.0	
Ever injectable contraception use				
Yes	54% (74/136)	47% (17/36)	57% (57/100)	0.31
No	46% (62/136)	53% (19/36)	43% (43/100)	
Current injectable contraception use				
Yes	53% (39/74)	53% (9/17)	53% (30/57)	0.90
No	47% (35/74)	47% (8/17)	47% (27/57)	
Level of condom use last month				
Did not have sex in past month	15% (21/136)	42% (15/36)	6% (6/100)	<0.001
Never	18% (24/136)	22% (8/36)	16% (16/100)	
Rarely	9% (12/136)	3% (1/36)	11% (11/100)	
Sometimes	18% (24/136)	8% (3/36)	21% (21/100)	
Frequently	10% (13/136)	3% (1/36)	12% (12/100)	
Always	31% (42/136)	22% (8/36)	34% (34/100)	
Worried about getting HIV				
Not at all worried	59% (80/136)	72% (26/36)	54% (54/100)	0.015
Somewhat worried	13% (17/136)	14% (5/36)	12% (12/100)	
Very worried	28% (38/136)	11% (4/36)	34% (34/100)	
No response/decline to answer	1% (1/136)	3% (1/36)	0% (0/100)	
Current risk reduction behaviours^a^				
Nothing	1.5% (2/136)	3% (1/36)	1% (1/100)	0.46
Abstinence (no sexual activity)	10% (13/136)	25% (9/36)	4% (4/100)	0.001
Have sex with only one partner	74% (101/136)	50% (18/36)	83% (83/100)	<0.001
Male/female condoms, one/all partners	84% (114/136)	72% (26/36)	88% (88/100)	0.04
Get tested for HIV	44% (60/136)	42% (15/36)	45% (45/100)	0.84
Other	3% (4/136)	8% (3/36)	1% (1/100)	0.46

a Multiple responses allowed.

### 3.1 Perceived risk of HIV

Perceived HIV risk differed significantly by region. More than a third of African participants, but just over 10% of US women, were very worried about HIV (*p *= 0.015). At baseline, most participants did something to reduce HIV risk, including having sex with only one partner (Africa vs. US, 83% vs. 50%, *p *< 0.001) and condom use (84%) – although only 31% overall said they always used condoms.

At exit, FGD participants continued to describe varying levels of perceived HIV risk. In Cape Town, participants felt that communities were less concerned about acquiring HIV than in the past, whereas in Harare, both knowledge and perceived risk were described as having increased.

*Haysuka! I don’t care about that (HIV). We will all have it; we’ll just go to the clinic and take our pills. It’s not a dog’s disease; it’s everyone’s disease. Participant, Cape Town*

US participants believed the risk was high, but that people were careless.

*It is a big concern, and I am actually afraid of the young people, because they are a little careless right now on a whole. I think they were born knowing about HIV and being a part of their lives. Not giving it much importance, as they should. They are not recognizing how serious this disease is. Participant, US*

### 3.2 Acceptability of injectable attributes

**Figure 1:**
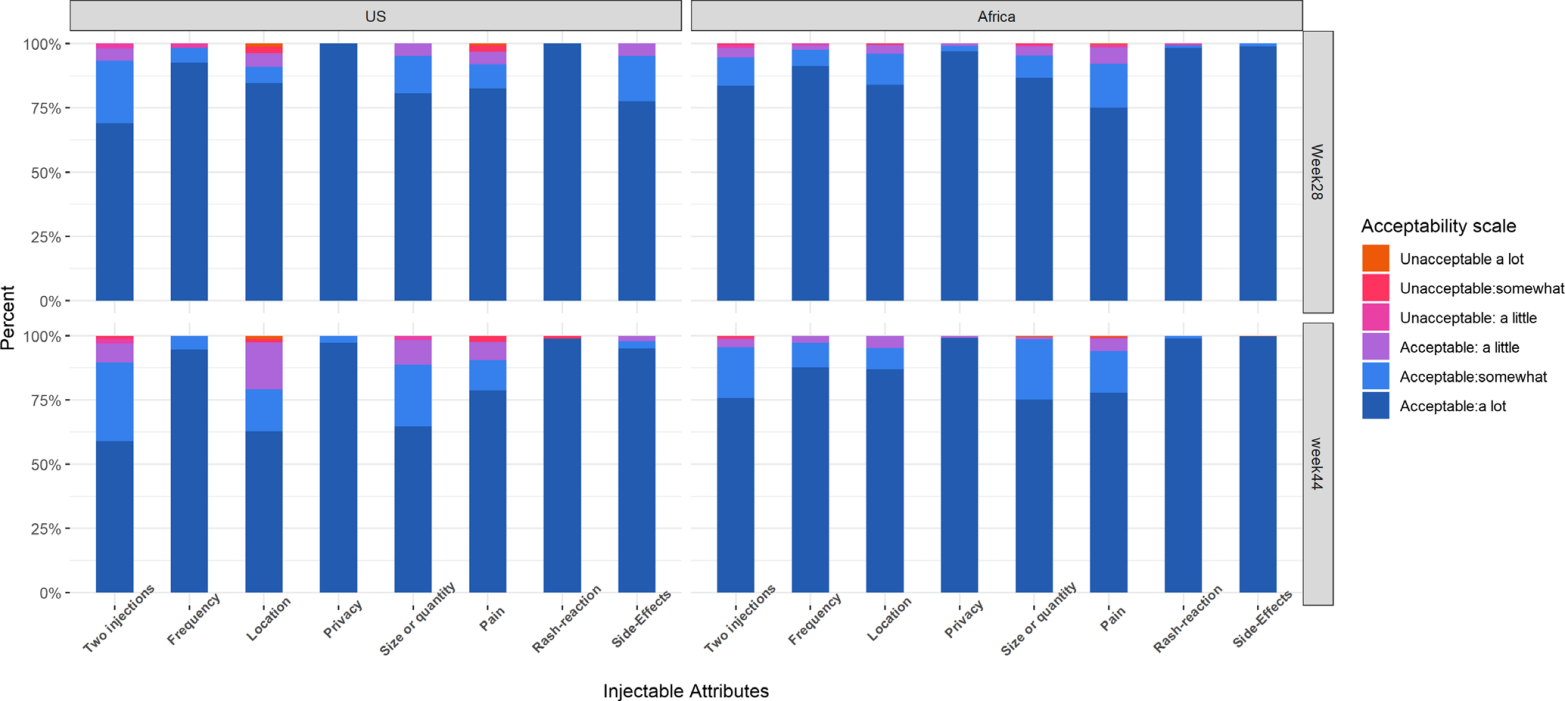
Acceptability of injectable attributes. *Among those who experienced any pain (n = 73 at Week 4, 68 at Week 28 and 66 at Week 44).

Although US participants reported lower acceptability of injectable attributes than did women from African sites, most participants rated injectable product attributes as highly acceptable (Figure 1). Acceptability ratings remained similar over time. Few participants reported rash or other side effects, but 56% to 67% reported pain with injection, with non‐significant differences over time and between arms. However, fewer than 15% reported pain to be a little, somewhat or very unacceptable at week 28 or 44.

Most participants (79%) reported at least one injection site reaction (ISR) up to their fourth injection at week 28, and 50% reported at least one ISR during injections five to six which occurred between week 28 and 44. The median number of ISRs for those reporting them was five to six, with as many as 18 different adverse events reported by at least one participant.

In FGDs, participants described initial fear of the injectable and variable experiences with pain over time. A US participant admitted:

*Getting the shots was my fear. Everything else I was fine with, the staff, everything. The atmosphere was just calm and welcoming. But I am scared of needles. I am 30 years old and two years ago you could not even take a butterfly needle to try and draw my blood until my mother got there.*

A Cape Town participant suggested that the pain experienced during her first injection almost prevented her from continuing:

*I felt like not coming back again. When I had my first injection, my butt was painful the whole week and so I thought to myself I’m not going to come back.*

### 3.3 Clinical trial context

Participants reported strong agreement with altruistic reasons for participating in the trial; the most highly endorsed reasons were to help the community and/or scientists. Women, especially in African settings, strongly agreed that they were seeking new experiences. Access to medical tests were somewhat endorsed, whereas concern about HIV risk for themselves and payment for participation were less endorsed. (Summary scores included in Table 1). Similarly, fewer than 15% of women reported any study‐related concerns.

In FGDs, women often described a complex decision‐making process for trial participation. They weighed concerns about trial procedures and product use, as well as partner or family opposition, with personal benefits from monetary reimbursements and access to HIV testing and other medical procedures, and to their families and communities in terms of new prevention products. The emphasis on these different factors could vary. While multiple women in the US and Cape Town sites agreed that initially they decided to participate “for the money,” most added that other factors were also important. Some described first becoming aware of these other health benefits during prior HIV prevention trial participation, like this US participant:

*I feel like I am giving back to the community, like I am part of a group that helped find preventative methods for HIV. I was part of other research studies. But, it is true, the few extra dollars are a motivation.*

Women in the Zimbabwe FGD, while also describing monetary and health‐related reasons, were more likely than those in the US and South African FGDs to mention altruistic reasons for joining the trial.

*I did not join for the sake of my life but for the sake of my fellows, such that even when I told my other relatives about it, they talked me out of it saying, ‘You will have side effects. You would die.’ and so forth. I gave them an example and said, ‘Isn’t it that when you were born, you were given those immunization injections?’ And she said, ‘Yes.’ And I then asked, ‘Where did they begin? You do not know where they started from, so it is just the same.’*

### 3.4 Interest in using an injectable PrEP product

At baseline, 56% of US participants and 81% of African participants preferred using a bi‐monthly injectable to other prevention methods, including daily oral pills, a vaginal ring or gel; interest increased in both regions over the study. At week 28, 79% of participants strongly endorsed the statement that they would “definitely use an injectable PrEP product for some time” if it were available in the future. Even more women (88%) strongly agreed that they would be “more interested in using an injectable if it was both for HIV and pregnancy prevention.” (Table 2). In a sensitivity analysis, there was no association between permanent discontinuation of the injectable and FIIP (data not shown.)

**Table 2:** Prevention preferences at baseline and 28 weeks

	Overall	US	Africa	*p*‐values
Baseline prevention preference	(n = 136)	(n = 36)	(n = 100)	0.02
No preference	0% (0/136)	0% (0/36)	0% (0/100)	
Bi‐monthly injection	74% (101/136)	56% (20/36)	81% (81/100)	
Daily oral pill	15% (20/136)	25% (9/36)	11% (11/100)	
Vaginal ring	4% (5/136)	8% (3/36)	2% (2/100)	
Vaginal gel	0% (0/136)	0% (0/36)	0% (0/100)	
Other (e.g. implant, sterilization, IUD)	7% (10/136)	11% (4/36)	6% (6/100)	
Follow‐up prevention preference (week 28)	(n = 113)	(n = 34)	(n = 79)	0.001
No preference	0% (0/113)	0% (0/34)	0% (0/79)	
Bi‐monthly injection	89% (101/113)	74% (25/34)	96% (76/79)	
Daily oral pill	10% (11/113)	24% (8/34)	4% (3/79)	
Vaginal ring	0% (0/113)	0% (0/34)	0% (0/79)	
Vaginal gel	1% (1/113)	3% (1/34)	0% (0/79)	
Other	0% (0/113)	0% (0/34)	0% (0/79)	
Week 28				
Would Definitely Use Injection	(n = 112)	(n = 32)	(n = 80)	0.001
A lot (disagree)	4% (5/112)	9% (3/32)	3% (2/80)	
Somewhat (disagree)	0% (0/112)	0% (0/32)	0% (0/80)	
A little (disagree)	3% (3/112)	6% (2/32)	1% (1/80)	
A little (agree)	5% (6/112)	13% (4/32)	3% (2/80)	
Somewhat (agree)	9% (10/112)	19% (6/32)	5% (4/80)	
A lot (agree)	79% (88/112)	53% (17/32)	89% (71/80)	
Would be more interested …if it was for both HIV and pregnancy prevention	(n = 113)	(n = 33)	(n = 80)	<0.001
A lot (disagree)	2% (2/113)	3% (1/33)	1% (1/80)	
Somewhat (disagree)	2% (2/113)	6% (2/33)	0% (0/80)	
A little (disagree)	1% (1/113)	3% (1/33)	0% (0/80)	
A little (agree)	3% (3/113)	6% (2/33)	1% (1/80)	
Somewhat (agree)	5% (6/113)	15% (5/33)	1% (1/80)	
A lot (agree)	88% (99/113)	67% (22/33)	96% (77/80)	

In longitudinal univariate models (Table 3), several of our theorized determinants, including acceptability of product attributes and of physical experiences, altruistic and personal motivations for trial participation, and HIV risk perception, were associated with FIIP. US participants reported significantly lower levels of FIIP than African women (OR = 0.14, *p* < 0.001) and those on TMC 278 had lower FIIP than those in the placebo arm (OR = 0.42, *p* = 0.04).

**Table 3:** Univariate and Multivariate model on Interest in Future Use of Injectable PrEP

Parameter	Comparison	Univariate Results				Multivariate Results			
		OR	LCL	UCL	*p*‐values	OR	LCL	UCL	*p*‐values
Product attributes		3.14	1.76	5.58	<0.001	2.38	1.32	4.28	0.004
Physical experiences		2.22	1.46	3.4	<0.001	1.32	0.6	2.92	0.48
Personal benefits		1.52	1.14	2	0.004	1.04	0.72	1.48	0.87
Altruism		3.36	1.62	6.98	0.001	1.74	0.64	4.68	0.27
Contraception use	Yes = 1 versus No = 0	1.24	0.62	2.48	0.54	1.34	0.64	2.84	0.45
Condom use		1.18	0.96	1.44	0.12	0.98	0.8	1.24	0.92
Worried HIV		2.26	1.4	3.64	<0.001	1.54	0.84	2.8	0.17
Treatment	Active = 1 versus Placebo = 0	0.42	0.18	0.96	0.04	0.56	0.22	1.44	0.23
Region	US = 1 versus Africa = 0	0.14	0.08	0.3	<0.001	0.26	0.1	0.66	0.005
Total ISR count		0.94	0.92	0.98	<0.001	0.98	0.96	1.02	0.39

LCL, lower confidence limit; OR, odds ratio; UCL, upper confidence limit.

In the longitudinal multivariate model, product attribute scores and being from a non‐US site remained the strongest predictors of FIIP. For example, in the adjusted model, a one unit increase in product attribute score over time increased the odds that a participant shows higher versus lower levels of agreement with FIIP more than twofold (OR = 2.38, *p* = 0.004). Total ISR count, treatment arm, perceived risk and risk behaviours were not significantly associated with FIIP, when controlling for other factors in the multivariate model (Table 3).

## 4 Discussion

Women’s challenges adhering to daily oral and vaginal PrEP within earlier clinical trials 14, 15, 16, and their relatively slow uptake of oral PrEP in service delivery settings 3, 27 underscore the need for alternative PrEP delivery modes. As new HIV prevention approaches move through clinical trial research, it is important to assess what features of these products are acceptable – even desirable, and for whom 28.

Several studies in African settings that assessed preferences among hypothetical or placebo PrEP products suggested that women’s acceptability of injectable products would be high, but that product choice and use patterns would also be shaped by individual and social context and geographies 28, 29. Although a decision was made not to further evaluate TMC 278 LA as a prevention agent, in part due to requirements for cold‐chain storage, this study provides one of the first opportunities to examine acceptability of an injectable PrEP product among a geographically diverse group of women using an active product. Its lessons may further complement those of another LAI PrEP product, Cabotegravir LA, which recently completed a phase 2 trial (HPTN 077) and is currently being evaluated among women in Africa and men in multiple settings. Like TMC 278, Cabotegravir LA is administered on a two‐month schedule, but does not require cold storage.

First, in the study, women’s acceptability of the injectable was high. In both the US and African contexts, women preferred an injectable over daily pills and vaginal rings or gel; this preference increased over time as women’s experience with the product increased. It is noteworthy that participants’ attitudes towards product attributes, including receiving two injections – one in each buttock every two‐months, was a stronger predictor of future interest in use than were their experiences with pain and other side effects. These findings are similar to acceptability data recently presented from a phase 2 trial of Cabotegravir LA (CAB LA) injectable (HPTN 077) 30 and a qualitative sub‐study of ÉCLAIR, a phase 2 trial of CAB LA in MSM, which found overall high levels of acceptability, despite frequent but variable experiences of pain 31. As additional long‐acting PrEP modalities like implants and antibodies move through clinical trial research 32, there is an opportunity to further assess how participants understand and experience product features and the relative trade‐offs they would consider in choosing and using a PrEP product.

Second, future interest in use of an injectable PrEP was significantly higher among African than US participants. This is not surprising, however, when considering regional differences in HIV prevalence. Indeed, almost half of African, but only one‐quarter of US participants perceived moderate to high HIV risk and reported low levels of consistent condom use. Nevertheless, US women of colour – especially in the northeast and south have increased risk of acquiring HIV 33. While PrEP awareness among US women remains low 4, 34, community‐based studies have identified a strong interest in PrEP use among US women 35, 36, 37 and globally 32, once women know about these prevention strategies.

The use of injectables for pregnancy prevention has increased dramatically in parts of eastern and South Africa 38. In 2015, injectables accounted for about 5% of contraceptive use globally, but upwards of 25% to 30% in parts of SSA 23. Women like the freedom from having to remember daily use and discretion afforded by injectable contraception 24, 39. Providers also seem to prioritize injectables in some countries over other methods, because they require less frequent clinic visits and can be provided by less skilled service providers 40, 41. Like preferences for injectable contraception, women in multiple settings have expressed preferences for an injectable PrEP product, which they perceived to last longer, require less medical intervention, have lower use burden and enable more discreet use 28, 42.

A third lesson relates to women’s strong interest in a dual‐purpose product for contraception and HIV prevention. This finding is in keeping with recent studies that have examined women’s preferences for multipurpose prevention technologies (MPTs) 43. One of the first MPT injectable regimens might involve the co‐administration of a single LAI PrEP injection with a contraceptive injection. Women’s acceptance of a two‐injection dosage in this study suggests that approach might be feasible.

The study has several limitations. First, acceptability of and adherence to products in randomized, placebo‐controlled trials is likely to differ from use of a known product in the context of daily life 44. As in past trials 45, 46, 47, our participants joined this study for many reasons, some unrelated to product use. But, while support received from trial sites to return on time for next injections encourages strong adherence, messages to participants about the product(s)’ unknown efficacy and the potential for adverse events may suppress acceptability.

Relatedly, although our study showed some association between HIV risk perception and acceptability of LAI PrEP, we did not collect detailed information about participants’ sexual behaviour or differences in perceived HIV risk by partner type. However, participants in this trial were generally older and at lower risk than women most likely to seek PrEP in real‐use contexts. Phase 3 trials, with larger samples of higher‐risk participants, can provide further information on characteristics of future LAI PrEP users. HPTN is not planning further studies of TMC 278 LA as a prevention agent, but two Phase 3 trials are in the field to assess the safety and efficacy of CAB LA compared to daily oral Truvada^®^ among HIV‐uninfected MSM (HPTN 083) and heterosexual women in SSA (HPTN 084). These trials should provide critical information about the relative acceptability of and adherence to oral and LAI PrEP among men and women in different settings.

Finally, this study did not assess the larger systems‐related factors that influence acceptability – especially access and cost. While programmes exist to provide oral PrEP free‐of‐charge for uninsured or low‐income individuals, the perceived high cost of PrEP medication and accessing coverage remain significant barriers in the US 23. Numerous PrEP demonstration projects are currently underway in SSA to evaluate strategies reaching and supporting PrEP use in different at‐risk populations, including MSM, female sex workers, HIV‐discordant couples, adolescents and young women 27, 48. Additional research is needed to determine how these systems‐related strategies might differ for provision of a LAI PrEP product, should CAB‐LA prove efficacious.

## 5 Conclusions

Although TMC 278 is not moving forward, this acceptability study provides valuable lessons for the successful introduction of future PrEP products more broadly. In this study, both US and African women reported an injectable PrEP product – TMC 278 LA – as highly acceptable and preferable to other new prevention products. Future interest in use was stronger among African than US women, who perceived higher levels of HIV risk. Women’s attitudes towards product attributes, influenced future interest in use more than pain experienced.

Given women’s and girls’ continuing burden of HIV infection globally and the need for – but challenges to achieving high levels of oral PrEP adherence, the development of a LAI PrEP or MPT product would provide an important new prevention tool.

## Competing interest

The authors declare no competing interests.

## Authors’ contributions

EET contributed to the design of acceptability measures and analyses and was primary author of final paper. SL and SZZ led statistical analyses. SP implemented quantitative data analysis. MA, PM and JS contributed to data collection and/or analysis of qualitative data. JJ, LGB, SS, JF and NS contributed to overall design and implementation of the study. All authors provided substantive review and comments leading to final manuscript.
